# The prescription pattern of initial treatment for type 2 diabetes in Beijing from 2011 to 2015

**DOI:** 10.1097/MD.0000000000014370

**Published:** 2019-02-22

**Authors:** Xiaowen Wang, Yaying Cao, Yao Wu, Chao Yang, Jing Song, Yaohua Tian, Mengying Wang, Man Li, Yiqun Wu, Yonghua Hu

**Affiliations:** a Department of Epidemiology and Biostatistics, School of Public Health; b Medical Informatics Center, Peking University Health Science Center, Beijing, China.

**Keywords:** hypoglycemic agents, prescription pattern, type 2 diabetes mellitus

## Abstract

The purpose of this study was to describe the prescription pattern of initial treatment for type 2 diabetes (T2DM) in Beijing from 2011 to 2015.

We selected 790,339 newly diagnosed outpatients with T2DM from the Beijing Medical Claim Data for Employees database between January 2011 and December 2015. The percentages of different treatments and agents were calculated from the patients’ 1st prescriptions. Subgroup analyses were conducted for primary, secondary, and tertiary hospital settings.

The initial treatments given to 57.7%, 30.7%, and 11.7% of patients were oral hypoglycemic agent (OHA) monotherapy, OHA polytherapy, and insulin, respectively. Alpha-glucosidase inhibitors (AGIs) (43.0%) were the most commonly used agents for monotherapy, followed by metformin (35.5%) and sulfonylureas (14.9%). AGIs were most commonly used in primary hospitals (52.0%), while metformin was prescribed most often in secondary (37.6%) and tertiary (41.8%) hospitals. From 2011 to 2015, there were increases in the use of AGIs (40.1–41.1%, *P* < .001) and metformin (34.0–40.4%, *P* < .001), but a decrease in the use of sulfonylureas (18.1–12.8%, *P* < .001). Similar trends were seen in the different hospital settings. Metformin plus an AGI, a sulfonylurea plus an AGI, and metformin plus a sulfonylurea were the most common OHA polytherapy combinations. The use of metformin plus an AGI increased from 13.8% in 2011 to 19.7% in 2015 (*P* < .001), while the use of a sulfonylurea plus an AGI, and metformin plus a sulfonylurea, did not change significantly.

Half of newly diagnosed patients with T2DM received an initial treatment of OHA monotherapy. Although the use of metformin increased from 2011 to 2015, both AGIs and metformin were the most commonly prescribed agents. The patterns differed from those of most other countries and identification of the underlying reasons will require further investigation.

## Abstract 2

Supplemental Digital Content is available in the text

## 1 Introduction

Type 2 diabetes mellitus (T2DM) is one of the most prevalent public health concerns worldwide. According to statistics from the International Diabetes Federation, in 2017 there were 425 million people living with diabetes worldwide, of whom 114.4 million patients were in China, which ranks 1st in the world.^[1]^ Among adults in China in 2013, the estimated overall prevalence of diabetes was 10.9% and that of prediabetes was 35.7%.^[2]^ T2DM accounted for more than 90% of all cases of diabetes.^[3]^ Patients with T2DM have higher risks of microvascular and macrovascular complications, leading to a high mortality rate and considerable medical costs.^[4,5]^ Tight glycemic control is favored for complication prevention, and pharmacologic agents are essential for most patients.^[6,7]^

The American Diabetes Association^[8]^ and the European Association for the Study of Diabetes^[9]^ have developed pharmacologic recommendations for T2DM intervention. For initial therapy, metformin is the preferred agent if tolerated and not contraindicated; dual therapy is considered in patients with hemoglobin A1c (HbA1c) ≥ 9%; and insulin therapy is recommended for those who are symptomatic and/or having HbA1c ≥ 10% and/or with a blood glucose level ≥ 300 mg/dL.^[8,10]^ Currently, 9 blood-glucose-lowering agents are used worldwide: biguanides, sulfonylureas, meglitinides, thiazolidinediones (TZDs), α-glucosidase inhibitors (AGIs), dipeptidyl peptidase-4 inhibitors (DPP-4i), sodium/glucose cotransporter 2 inhibitors, insulins, and glucagon-like peptide-1 receptor agonists. Multiple treatment options have also been proposed in guidelines from different regions.^[8,9,11–13]^ Accordingly, the initial prescription patterns of hypoglycemic medicines differ by region.^[14–19]^ For example, metformin was the most prevalent initial choice in the United States in 2012, accounting for 90.8% of oral antidiabetic drug (OAD) monotherapy,^[15]^ while in Italy metformin accounted for 68.0%.^[16]^ Overbeek et al analyzed data from five European countries, and observed a sharp discrepancy in hypoglycemic treatment even among neighboring countries, which might reflect differences in the screening and management patterns of these countries.^[14]^

Despite China having the largest number of patients with T2DM worldwide, few studies have described the prescription patterns of hypoglycemic agents there. Lu et al reported higher use of the newer, more expensive agents of every class of antidiabetic medication in China compared with Brazil or Thailand.^[20]^ In 2010, the DiaSTAGE study in China showed that, in current antidiabetic treatment regimens, insulin secretagogues such as sulfonylureas or glinides were the most common OADs, followed by metformin.^[21]^ To the best of our knowledge, the initial treatment pattern of hypoglycemic agents in China has not been described previously. An accurate description of the initial treatment pattern would provide valuable information for the management of T2DM and the establishment of health policies. Electronic administrative databases are ideal, reliable sources of data for investigating prescription patterns in a large population.^[22]^ Using an administrative database in Beijing, we conducted this study to describe the prescription pattern of initial treatment for newly diagnosed patients with T2DM.

## 2 Methods

### 2.1 Data source

All data were obtained from the Beijing Medical Claim Data for Employees (BMCDE), which was described in detail in our previous studies.^[22,23]^ Overall, the database recorded the reimbursement information of all working and retired employees in Beijing from 2006 to 2015, covering 9 million beneficiaries at the end of 2015. Information from the same patient over time could be linked anonymously using an encrypted patient code. Anonymized information regarding patient demographic characteristics (age and sex), clinical diagnoses (International Classification of Disease edition 10 [ICD-10] and descriptive texts), and details on dispensed medications (branded and generic drug names, formulations, fees, and dispensing dates) were included. Our use of encrypted retrospective information did not require ethics approval.

### 2.2 Study population

From the source population, we identified all outpatients who were newly diagnosed with T2DM (ICD-10 codes: E11-E14) between January 1, 2011 and December 31, 2015. The date of diagnosis of T2DM was defined as the index date. Only patients without T2DM or prescription of hypoglycemic agents during the 12 months preceding the index date were included. Patients who were diagnosed with type 1 diabetes or gestational diabetes, or subjects with any missing demographic, clinical or medication data, were excluded.

The study patients were stratified into 3 main categories according to their 1st prescription of a hypoglycemic agent: oral hypoglycemic agent (OHA) monotherapy (patients with prescriptions of a single OHA), OHA polytherapy (patients with prescriptions of 2 or more OHAs or a fixed combination), and insulin therapy (patients with prescriptions of insulin with or without an OHA). Since the data set covered the years of 2006 to 2015, we extracted the comorbidities of each patient that had been recorded for a minimum of 5 years before the index date, which were identified as follows: hypertensive diseases (ICD-10 codes: I10-I15), cerebrovascular diseases (ICD-10 codes: I60-I69), ischemic heart diseases (ICD-10 codes: I20-I25), liver diseases (ICD-10 codes: K70-K77), and renal failure (ICD-10 codes: N17-N19).

### 2.3 Drug classification

Hypoglycemic agents were sorted according to the Anatomical Therapeutic Chemical classification system (World Health Organization, version 2018^[24]^), and included A10A (insulin and analogues), A10BA (biguanides [metformin]), A10BB (sulfonylureas), A10BF (AGIs), A10BG (TZDs), A10BX (other blood-glucose lowering drugs), and A10BD (fixed combination blood-glucose lowering agents).

### 2.4 Statistical analysis

The baseline demographic characteristics of patients are shown as means (and standard deviations) for continuous variables, and as numbers (and percentages) for categorical variables. The 1st prescription of a hypoglycemic agent(s) for each patient was selected to calculate the percentages of different classes of agents used for each study year. The Cochran–Armitage trend test was used to assess the statistical significance of prescribing patterns from 2011 to 2015. One-way analysis of variance and Chi-squared tests were used to assess the statistical significance of patient characteristics. The results for patients treated in primary, secondary, and tertiary hospitals are reported separately. All statistical tests were 2-tailed, and a *P*-value of <.05 was considered statistically significant. All analyses were conducted using the R programming language (V.3.2.2, R Development Core Team).

## 3 Results

### 3.1 Characteristics of the study patients

During the study period, 790,339 newly diagnosed outpatients with T2DM were identified, of whom 48.9% were men. The average age was 56.1 years. Most (60.4%) of the outpatients had at least 1 comorbidity. Diagnoses of 37.5%, 26.2%, and 36.3% of the patients were made in primary, secondary, and tertiary hospitals, respectively. Details of the basic characteristics of the study patients are shown in Table 1.

**Table 1:**
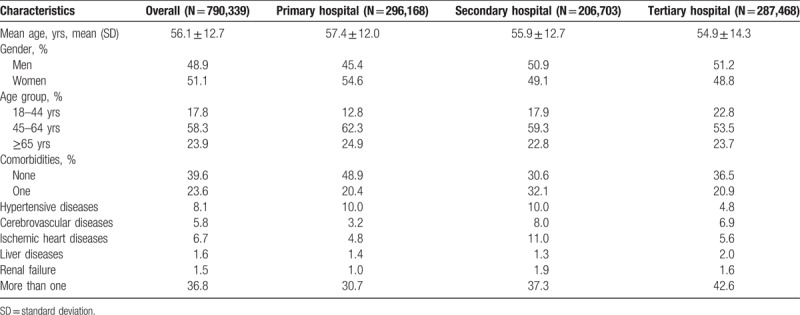
Characteristics of the study patients.

### 3.2 Three therapy groups

The OHA monotherapy, OHA polytherapy, and insulin were given as initial treatment to 57.7%, 30.7%, and 11.7% of the patients, respectively. Those who received OHA monotherapy as initial treatment were more likely to be female (female: 58.4%; male: 50.4%), older than 45 years (18–44: 52.3%; 45–65: 55.2%; ≥65: 54.3%), and with comorbidities (without comorbidities: 52.8%; with 1 comorbidity: 56.3%; with more than 1 comorbidity: 56.7%).

The OHA monotherapy was the most prevalent initial treatment in all 3 levels of health care setting, accounting for 64.7% in primary hospitals, 51.9% in secondary hospitals, and 47.7% in tertiary hospitals. The prescribing trend described above was consistent in all 3 levels of health care setting, that is, patients who were female, older than 45 years, and with comorbidities were more likely to be given OHA monotherapy as their initial treatment (see Table, Supplemental Digital Content 1, which illustrates the 3 patient groups stratified according to the 1st prescription).

**Figure 1:**
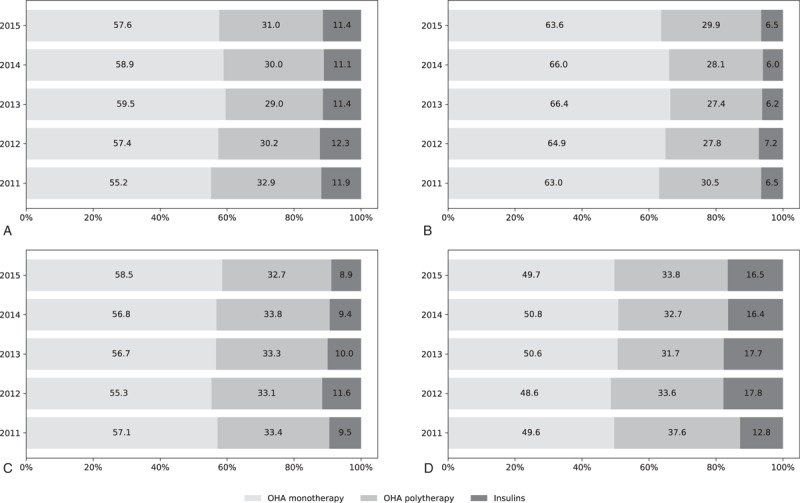
Percentage of patients in the three therapy groups from 2011 to 2015: overall (A), patients in primary hospitals (B), secondary hospitals (C), and tertiary hospitals (D). The X-axis shows the year. The Y-axis shows the percentage of patients treated with a specific class of the total patients treated with any antidiabetic medication. OHA = oral hypoglycemic agents.

From 2011 to 2015, the percentage of patients initialing their treatment with OHA monotherapy increased slightly from 55.2% in 2011 to 57.6% in 2015 (*P* < .001). There was a slight decrease in the use of OHA polytherapy from 32.9% in 2011 to 31.0% in 2015 (*P* < .001), while the percentage of patients receiving insulins remained stable, fluctuating at about 11.5% (Fig. 1A). Similar trends were seen in primary and secondary hospitals (Fig. 1B, C). In tertiary hospitals, the percentages of patients who were prescribed OHA monotherapy remained stable, while those who received OHA polytherapy decreased from 37.6% in 2011 to 33.8% in 2015 (*P* < .001), and those who received insulins increased from 12.8% in 2011 to 16.5% in 2015 (*P* < .001) (Fig. 1D).

### 3.3 Specific OHA categories for monotherapy

For OHA monotherapy, AGIs were the most frequently used agents, accounting for 43.0%. Metformin and sulfonylureas ranked 2nd and 3rd, accounting for 35.5% and 14.9%, respectively. With regard to the different clinical settings, AGIs were the most prevalent agents in primary hospitals (52.0%), while metformin was the most prevalent in secondary (37.6%) and tertiary (41.8%) hospitals.

**Figure 2:**
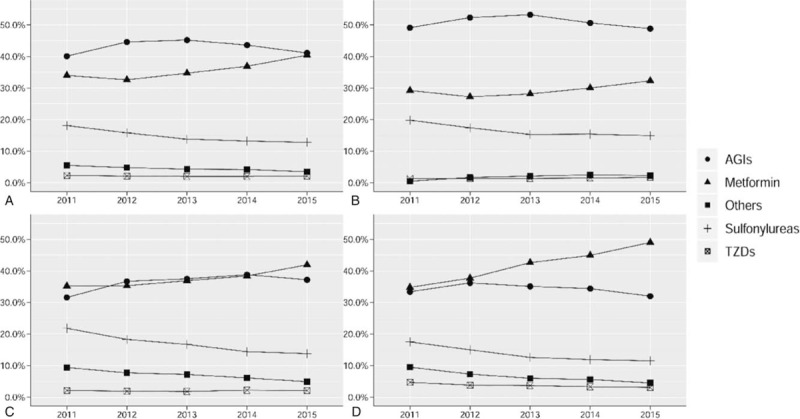
Percentage of patients who received a specific class of oral hypoglycemic agent as monotherapy from 2011 to 2015: overall (A), in primary hospitals (B), in secondary hospitals (C), and in tertiary hospitals (D). The X-axis shows the year. The Y-axis shows the percentage of patients treated with a specific monotherapy class of the total patients treated with any monotherapy. AGIs = α-glucosidase inhibitors, TZDs = thiazolidinediones.

From 2011 to 2015, the use of AGIs increased from 40.1% in 2011 to 45.2% in 2013, then decreased to 41.1% in 2015 (*P* < .001). Metformin increased successively from 34.0% in 2011 to 40.4% in 2015 (*P* < .001). Meanwhile, the percentage of patients treated with sulfonylureas decreased from 18.1% in 2011 to 12.8% in 2015 (*P* < .001) (Fig. 1, panel A). Similar trends for specific categories of OHA monotherapy were seen across the 3 levels of hospital setting (Fig. 2, panels B–D).

### 3.4 Specific OHA categories for polytherapy

For OHA polytherapy, the top 3 most prescribed combinations were metformin plus an AGI, a sulfonylurea plus an AGI, and metformin plus a sulfonylurea, accounting for 16.9%, 16.4%, and 15.6%, respectively. The top 3 combinations were the same in all 3 hospital settings, accounting for about 50% of all OHA polytherapy prescriptions.

**Figure 3:**
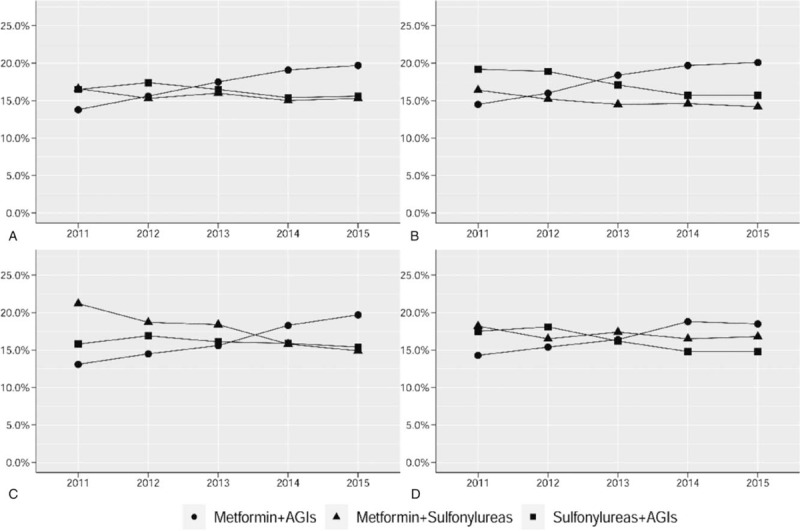
Percentage of patients who received the top three combinations of oral hypoglycemic agent polytherapy from 2011 to 2015: overall (A), in primary hospitals (B), in secondary hospitals (C), and in tertiary hospitals (D). The X-axis shows the year. The Y-axis shows the percentage of patients treated with a specific combination of the total patients treated with any polytherapy. AGIs = α-glucosidase inhibitors.

The use of metformin plus an AGI increased from 13.8% in 2011 to 19.7% in 2015 (*P* < .001), while the use of a sulfonylurea plus an AGI, and metformin plus a sulfonylurea, did not change significantly (Fig. 3A). The use of metformin plus an AGI increased significantly in all 3 hospital settings. The use of a sulfonylurea plus an AGI decreased most in primary hospitals, while the use of metformin plus a sulfonylurea decreased most in secondary hospitals (Fig. 3B–D).

## 4 Discussion

To optimize blood glucose levels and prevent diabetes complications, medicine is essential for most patients with T2DM.^[6,7]^ Given the differences in health care policy and diabetes screening and management patterns, there is diversity in prescription patterns for hypoglycemic agents among different countries.^[14–18,25]^ Considering that there are few reports on the initial treatment pattern for T2DM in China, we chose to conduct this study using a population-based administrative database (BMCED), a reliable source for investigating prescription patterns in a large population such as that in Beijing.^[22]^

More than half of patients started their treatment with 1 OHA, and this percentage increased slightly over the study period. The prescription pattern for OHA monotherapy as the dominant player in newly diagnosed T2DM was similar to that the United States (56.7% of patients in 2012^[15]^) and Denmark (62% of patients in 2010–2013^[19]^), indicating that most outpatients tended to be in mild or moderate condition. However, approximately 11% of patients were initially prescribed an insulin, probably reflecting the large number of people with severe hyperglycemia, even among those newly diagnosed. Insulins should be considered when hyperglycemia is severe.^[12]^ Medical education and blood glucose monitoring in high-risk people is necessary for the early detection of hyperglycemic status.

As for specific drugs in monotherapy, AGIs were persistently the most commonly prescribed, while metformin ranked 2nd. This was in contrast to other countries where metformin has played the dominant role, accounting for 50% to 90%.^[20,25,26]^ According to the most recent Chinese guidelines,^[11]^ AGIs are also recommended for initial therapy, based on evidence from randomized clinical trials that showed that acarbose was similar to metformin in efficacy for Chinese patients with newly diagnosed T2DM.^[27,28]^ Acarbose retarded intestinal carbohydrate digestion and absorption, especially targeting postprandial hyperglycemia. Hence has been shown to be superior in Chinese populations because they consume a higher proportion of carbohydrates compared with the standard American diet.^[29,30]^

The use of metformin increased from 2011 to 2015, especially in tertiary hospitals, accompanied with decreasing trends in the use of sulfonylureas, TZDs, and other agents. These trends were similar to those of other countries. It has been reported that the usage of metformin increased from 74.7% of OAD monotherapy users in 2007 to 90.8% in 2012, whereas sulfonylurea usage decreased in five European countries as well as the United States.^[14,15]^ In China, this trend may be partly attributable to health care reforms that aim to reduce the reliance on pharmaceutical sales as revenue sources for hospitals.^[31]^ Rosiglitazone and pioglitazone were the top 2 TZDs used in China. During the study period, the percentage of patients using TZDs dropped, which was similar in the United States, some countries in Europe, and Australia,^[14,15,25,32]^ given the increasing risk of cardiovascular diseases with rosiglitazone^[33]^ and the bladder cancer warnings for pioglitazone.^[34]^

In terms of polytherapy, metformin plus a sulfonylurea, a sulfonylurea plus an AGI, and metformin plus an AGI were the top 3 combinations, accounting for <20%. In the United States and Korea, metformin plus a sulfonylurea is the dominant combination, accounting for 31% and 41.7%, respectively.^[35,36]^ This difference might reflect the different prescription patterns between countries. In China, besides metformin, AGIs, and sulfonylureas were the top 2 choices for hypoglycemic control.

We analyzed the prescription patterns at the 3 levels of hospital for potential differences. The percentages of patients who were started on OHA monotherapy were higher in primary hospitals (64.7%) than in secondary (51.9%) and tertiary (47.7%) hospitals. Although they showed the same temporal trend, AGIs ranked 1st for monotherapy in primary hospitals, while metformin ranked 1st in secondary and tertiary hospitals, a discrepancy that might reflect the different patient features at each hospital setting. Among the patients in the BMCED, the percentages of females and patients over 45 years of age were higher in primary hospitals, and the percentage of patients with more comorbidities was higher in tertiary hospitals. Patient preferences might result in sex differences in the treatment schemes given to patients with T2DM in different levels of hospitals, which were also observed in previous studies.^[37,38]^ AGIs represented a viable option for older adult patients with T2DM because they offer significant reductions in glycemia but without serious hypoglycemia, weight gain, and cardiovascular events.^[39]^ Meanwhile, the use of metformin in patients with more comorbidities (including cardiovascular, liver, or renal diseases) was associated with improvements in key clinical outcomes.^[40]^ Furthermore, apart from the differences in patient features, the availability of different antidiabetic drugs, physician behaviors, and prescribing policies were all different,^[41,42]^ and may have contributed to the discrepancy between hospitals. Prescribing patterns could be influenced by national medicine policies, which may in turn serve as guidance for future policymaking. Policymakers could improve future policies using current evidence to ensure the same availability of antidiabetic drugs at all hospitals as well as their rational use by health care providers and patients.^[43]^

There were limitations in our study. First, the BMCDE database only included those patients who were employed and covered by basic medical insurance, and would differ from unemployed, uninsured patients across a range of characteristics. Caution should be paid when generalizing these results to other populations. Second, because medical claim data and other important clinical information, such as blood glucose levels, and hepatic and renal function status, were absent from the BMCDE database, an intensive analysis of the data was not possible. Furthermore, prescription patterns are associated with the medical insurance system of a country. Hence, we failed to analyze some new classes of antidiabetic medication (e.g., DPP-4i was not included in China Health Insurance Coverage until 2017^[44]^); however, in middle-income countries, conventional antidiabetic drugs are likely to be more affordable than newer agents.^[45]^

## 5 Conclusion

Our study depicted the prescription pattern for initial treatment in patients with T2DM in Beijing within a large employed population. About half of newly diagnosed patients with T2DM received an initial treatment of OHA monotherapy. Although the use of metformin increased from 2011 to 2015, both AGIs and metformin were the most commonly used OHA agents. Prescription patterns varied among the primary, secondary, and tertiary hospital settings, likely owing to different medical approaches. The prescription pattern in China was different from that of most other countries, where metformin is the dominant OHA. More precise studies are needed to identify the underlying reasons for the unique prescription pattern of initial T2DM treatment in China.

## Author contributions

**Conceptualization:** Yiqun Wu, Yonghua Hu.

**Data curation:** Yonghua Hu.

**Formal analysis:** Xiaowen Wang, Yaying Cao, Yao Wu, Chao Yang, Yiqun Wu, Yonghua Hu.

**Investigation:** Xiaowen Wang.

**Supervision:** Yiqun Wu.

**Validation:** Yiqun Wu.

**Writing – original draft:** Xiaowen Wang, Yaying Cao, Yao Wu, Chao Yang, Jing Song, yaohua Tian, Mengying Wang, Man Li, Yiqun Wu.

**Writing – review & editing:** Xiaowen Wang, Yiqun Wu, Yonghua Hu.

## Supplementary Material

Supplemental Digital Content
