# Supplementary material for: The prescription pattern of initial treatment for type 2 diabetes in Beijing from 2011 to 2015
Source: Medicine (Baltimore). 2019 Feb 22;98(8):e14370. doi: 10.1097/MD.0000000000014370 (PMC6408070; doi:10.1097/MD.0000000000014370)
Supplement: Supplemental Digital Content [file medi-98-e14370-s001.doc]

**Supplemental Digital Content 1. Table that illustrates three therapy groups for patients according to their first prescriptions, %.**

|  | Primary Hospitals | | |  | Secondary Hospitals | | |  |  | Tertiary Hospitals |  |
| --- | --- | --- | --- | --- | --- | --- | --- | --- | --- | --- | --- |
|  | OHA  Monotherapy | OHA  Poly-therapy | Insulin |  | OHA  Monotherapy | OHA  Poly-therapy | Insulin |  | OHA  Monotherapy | OHA  Poly-therapy | Insulin |
| Overall | 64.7 | 29.5 | 6.0 |  | 51.9 | 38.4 | 10.0 |  | 47.7 | 36.1 | 16.2 |
| Gender |  |  |  |  |  |  |  |  |  |  |  |
| Male | 62.4 | 31.2 | 6.4 |  | 48.0 | 41.1 | 10.9 |  | 42.5 | 40.3 | 17.3 |
| Female | 66.7 | 27.8 | 5.5 |  | 55.2 | 35.7 | 9.1 |  | 52.1 | 32.8 | 15.1 |
| Age (yrs.) |  |  |  |  |  |  |  |  |  |  |  |
| 18-44 | 61.5 | 29.9 | 8.7 |  | 49.2 | 38.1 | 12.8 |  | 44.3 | 33.2 | 17.5 |
| 45-64 | 64.7 | 29.7 | 5.6 |  | 52.0 | 39.4 | 8.6 |  | 47.6 | 40.0 | 12.7 |
| ≥65 | 66.5 | 28.2 | 5.3 |  | 52.2 | 36.4 | 11.4 |  | 49.3 | 32.8 | 22.9 |
| Comorbidities |  |  |  |  |  |  |  |  |  |  |  |
| None | 63.3 | 31.1 | 5.6 |  | 49.4 | 41.4 | 9.2 |  | 45.0 | 27.1 | 13.9 |
| One | 60.2 | 34.7 | 5.1 |  | 53.2 | 36.0 | 10.8 |  | 45.1 | 39.1 | 15.8 |
| More than one | 67.2 | 25.8 | 7.0 |  | 54.5 | 35.2 | 10.3 |  | 51.3 | 30.3 | 18.4 |
